# Identification of Novel Prognostic Signatures for Clear Cell Renal Cell Carcinoma Based on ceRNA Network Construction and Immune Infiltration Analysis

**DOI:** 10.1155/2022/4033583

**Published:** 2022-03-14

**Authors:** Lu Zhou, Juan Ye, Fengyun Wen, Hong Yu

**Affiliations:** ^1^Department of Radiation Oncology, Cancer Hospital of China Medical University, Liaoning Cancer Hospital & Institute, No. 44 Xiaoheyan Road, Dadong District, Shenyang, 110042 Liaoning, China; ^2^Department of Radiology, Suzhou Kowloon Hospital, Shanghai Jiaotong University School of Medicine, No. 118 Wansheng Street, Suzhou Industrial Park, Suzhou, 215028 Jiangsu, China

## Abstract

**Objective:**

Clear cell renal cell carcinoma (ccRCC) carries significant morbidity and mortality globally and is often resistant to conventional radiotherapy and chemotherapy. Immune checkpoint blockade (ICB) has received attention in ccRCC patients as a promising anticancer treatment. Furthermore, competitive endogenous RNA (ceRNA) networks are crucial for the occurrence and progression of various tumors. This study was aimed at identifying reliable prognostic signatures and exploring potential mechanisms between ceRNA regulation and immune cell infiltration in ccRCC patients.

**Methods and Results:**

Gene expression profiling and clinical information of ccRCC samples were obtained from The Cancer Genome Atlas (TCGA) database. Through comprehensive bioinformatic analyses, differentially expressed mRNAs (DEmRNAs; *n* = 131), lncRNAs (DElncRNAs; *n* = 12), and miRNAs (DEmiRNAs; *n* = 25) were identified to establish ceRNA networks. The CIBERSORT algorithm was applied to calculate the proportion of 22 types of tumor-infiltrating immune cells (TIICs) in ccRCC tissues. Subsequently, univariate Cox, Lasso, and multivariate Cox regression analyses were employed to construct ceRNA-related and TIIC-related prognostic signatures. In addition, we explored the relationship between the crucial genes and TIICs via coexpression analysis, which revealed that the interactions between MALAT1, miR-1271-5p, KIAA1324, and follicular helper T cells might be closely correlated with the progression of ccRCC. Ultimately, we preliminarily validated that the potential MALAT1/miR-1271-5p/KIAA1324 axis was consistent with the ceRNA theory by qRT-PCR in the ccRCC cell lines.

**Conclusion:**

On the basis of the ceRNA networks and TIICs, we constructed two prognostic signatures with excellent predictive value and explored possible molecular regulatory mechanisms, which might contribute to the improvement of prognosis and individualized treatment for ccRCC patients.

## 1. Introduction

Renal cell carcinoma (RCC) is one of the most common malignancies of the urological system [1]. Clear cell renal cell carcinoma (ccRCC) is the most common type of malignant renal tumor with more malignant features and poor prognosis, accounting for most RCC-associated deaths [2]. Therapeutically, ccRCC patients generally lack sensitivity to conventional chemotherapy and radiotherapy. Although targeted therapies have made significant progress in improving the survival of patients with ccRCC, the median survival rate remains poor for high-grade and advanced-stage patients. Therefore, to improve the prognosis of ccRCC patients, the exploration of novel mechanisms involved in ccRCC development and the identification of potential prognostic markers and therapeutic targets are urgently required.

Salmena et al. presented the competing endogenous RNA (ceRNA) hypothesis, whereby lncRNA is able to compete with mRNA to combine with miRNA through miRNA response elements (MREs), thereby depressing miRNA-induced downregulation of their target mRNAs [3]. Numerous studies have demonstrated that ceRNA regulatory networks play an essential role in cancer development [4]. Several researches in recent years have shown that immune cell infiltration in the tumor microenvironment (TME) could be involved in the antitumor immune response. RCC is considered an immunogenic tumor on the basis of the high level of tumor-infiltrating T cells, the incidence of spontaneous tumor regression, and the response to immunotherapy [5, 6]. Furthermore, growing evidence indicated that ceRNA networks regulate the crosstalk between tumor cells and tumor-infiltrating immune cells (TIICs). Immune cell infiltration is the basis for effective immunotherapy, which plays a crucial role in the prognosis of various malignancies [7]. Thus, exploring markers to assess immune infiltration and the potential regulatory mechanisms will contribute to the advancement of immunotherapy researches. Up to now, the role of mechanisms regulating immune cell infiltration through gene regulatory networks and tumor cell-intrinsic signaling pathways is not clear in ccRCC.

In the present study, differentially expressed ceRNAs were identified on the basis of the transcriptome data of ccRCC in TCGA database. We assessed the proportion of TIICs in ccRCC using the CIBERSORT algorithm. Subsequently, two prognostic risk signatures were constructed according to survival-related ceRNAs and TIICs. Besides, we innovatively identified the potential immune-related biomarkers in ccRCC based on coexpression analysis between ceRNAs and TIICs.

## 2. Materials and Methods

### 2.1. Data Source and Differential Expression Analysis

The gene expression profiles of the ccRCC and normal samples were collected from TCGA database (https://portal.gdc.cancer.gov/). Meanwhile, the relevant clinical information was obtained from TCGA database as well. Patients with less than 30 days of follow-up and incomplete clinical information were removed from the survival analysis. The R package DEseq2 was used to identify differentially expressed mRNAs (DEmRNAs), lncRNAs (DElncRNAs), and miRNAs (DEmiRNAs) after eliminating non-ccRCC specific expression genes which were not detected in either tumor or normal groups. The logFC (fold change) > 1.0 or <−1.0 and false discovery rate (FDR) adjusted *P* value < 0.05 were exploited as the filtering criteria.

### 2.2. Construction of ceRNA Networks

A ceRNA network was established by GDCRNATools package in R software [8]. The interactive relations in both DEmiRNA-DEmRNA and DElncRNA-DEmiRNA were derived from StarBase (http://mirtarbase.mbc.nctu.edu.tw/). The miRNAs with significant outcomes in hypergeometric testing and correlation analysis were singled out to construct ceRNA networks. Ultimately, ceRNA networks were visualized using Cytoscape version 3.4.0 [9].

### 2.3. Functional Enrichment Analysis

Gene Ontology (GO) function analysis and Kyoto Encyclopedia of Genes and Genomes (KEGG) pathway enrichment analysis of DEmRNAs in the ceRNA network were realized via the R package clusterProfiler [10]. GOplot and enrichplot packages in R software were utilized to visualize the results of GO function analysis and KEGG pathway enrichment analysis, respectively. The cut-off criterion was *P* value < 0.05.

### 2.4. Survival Analysis and Nomograms of the Pivotal Genes in ceRNA Networks

Firstly, the Cox regression analysis and Lasso regression analysis were performed for all members of the ceRNA network to screen the significant variables in the initial Cox models and ensure that the multifactor models were not overfitting. Subsequently, based on the multivariable models, a nomogram was formulated to predict the risk score for each patient's overall survival (OS). Ultimately, to assess the discrimination and accuracy of the nomogram, receiver operating characteristic (ROC) curves and calibration curves were performed. Meanwhile, Kaplan-Meier (K-M) survival analysis was employed to explore the survival variations between high-risk and low-risk groups.

### 2.5. Estimation of TIIC Abundance

CIBERSORT is an analytical tool to precisely estimate fractions of multiple human immune cells in gene expression profiles. The superior performance of CIBERSORT aroused an increasing focus on cell heterogeneity studies [11, 12]. Our current study calculated the proportion of 22 types of TIICs in ccRCC tissues through the CIBERSORT algorithm. The samples could be used for further survival studies only when the threshold of *P* value was less than 0.05. Then, the barplot and heat map were drawn to describe the composition of TIICs in each sample. In addition, the differences of TIICs between tumor and matched normal tissues were assessed using the Wilcoxon rank-sum test, and the results were visualized by the R package vioplot.

### 2.6. Survival Analysis and Nomograms of the Crucial TIICs

Univariate Cox regression analysis was carried out to investigate the prognostic TIICs. Lasso regression, as a dimensionality reduction analysis, was utilized to filter TIICs. Subsequently, multivariable Cox regression analysis was performed to construct a TIIC-related prognostic signature. K-M survival and ROC curves were conducted to evaluate the predictive performance for prognostic signature. Meanwhile, a nomogram was construed to predict the prognosis of each ccRCC patient, and a calibration curve was applied to assess the discrimination and accuracy. Ultimately, we investigated the association between significant ceRNAs and TIICs based on the Pearson correlation analysis.

### 2.7. Cell Culture and Transfection

The human ccRCC cell lines (786-O, ACHN, Caki-1, and OSRC-2) and the normal renal tubular epithelial cell line (HK-2) were obtained from the Cell Bank of the Chinese Academy of Sciences (Shanghai, China), which were cultured according to the suppliers' instructions, respectively. All experiments were carried out with cells in the exponential phase. Small interfering RNAs (siRNAs), si-MALAT1 and si-NC, were provided by RiboBio (Guangzhou, China) and were transiently transfected into Caki-1 and OSRC-2 cell lines using jetPRIME® (Invitrogen, Polyplus-transfection® SA, France) according to the manufacturer's protocol.

### 2.8. Quantitative Real-Time PCR (qRT-PCR)

Total RNA was extracted from the cultured cells with the Trizol reagent (Invitrogen, CA, USA). Afterward, RNAs were reversely transcribed into cDNA with GoScript™ Reverse Transcription Mix (for mRNA and lncRNA, Promega, America) or miScript II RT Kit and miScript SYBR® Green PCR Kit (for miRNA, QIAGEN, Germany). qRT-PCR was conducted with SYBR® Premix Ex Taq™ II (Takara, Japan) and measured in Applied Biosystems® 7500 Real-Time Systems (Thermo Fisher, IL, USA). 18S and U6 were used as the normalization control for mRNA and miRNA RCR, respectively. The RNA relative expression was calculated by the 2^−ΔΔCt^ method. The primers for amplification of targets are shown in Supplementary Table 2.

### 2.9. Statistical Analysis

Only a two-sided *P* value < 0.05 was considered statistically significant. All statistical analyses were performed with R version 4.0.3 software (package: GDCRNATools DEseq2, edgeR, ggplot2, clusterProfiler, glmnet, preprocessCore, survminer, survival, timeROC, rms, pheatmap, corrplot, and vioplot).

## 3. Results

### 3.1. Identification of ccRCC-Related Differentially Expressed Genes

**Figure 1:**
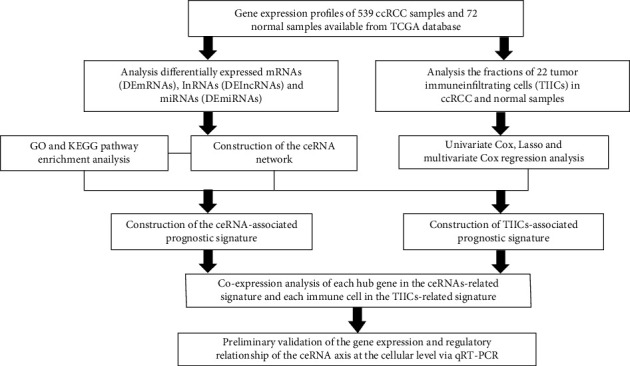
The flow diagram of the analytical procedure.

**Figure 2:**
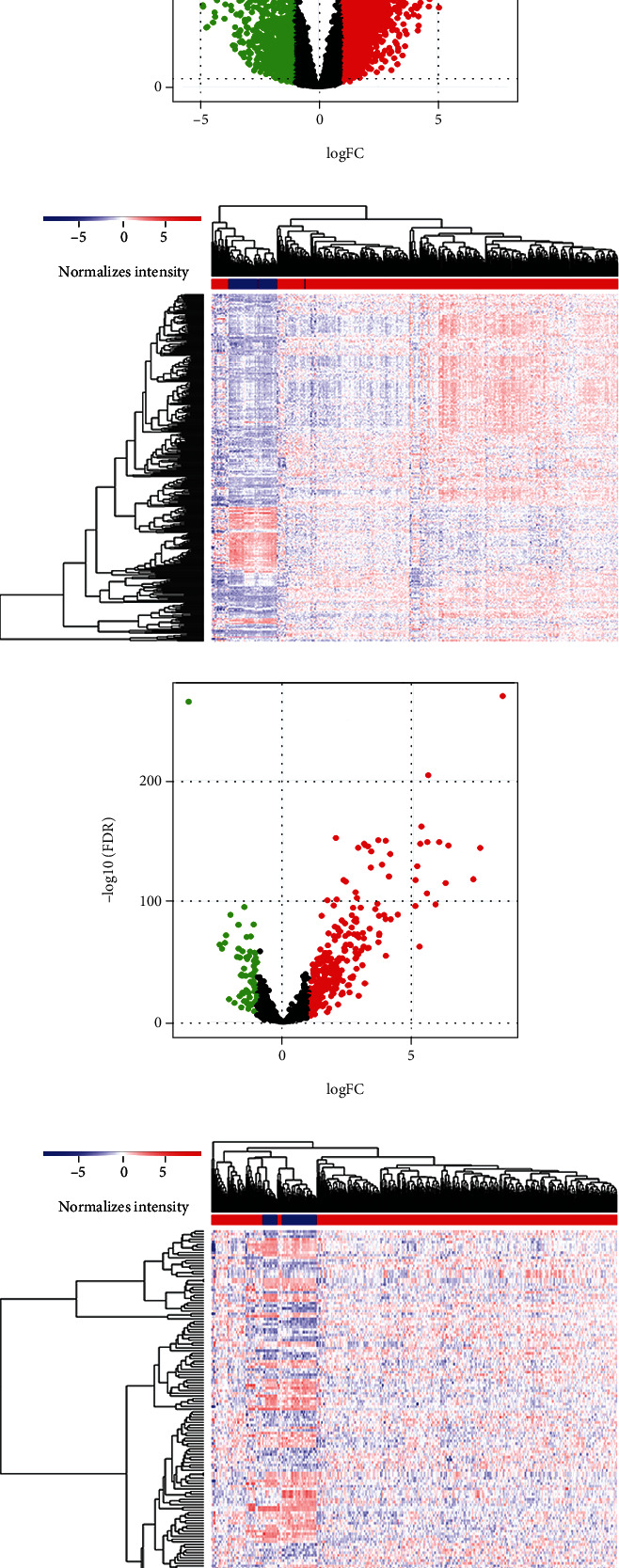
The differentially expressed genes between ccRCC and normal tissues. (a–f) The heat map (a) and the volcano plot (b) of 3075 DEmRNAs; the heat map (c) and the volcano plot (d) of 359 DElncRNAs; the heat map (e) and the volcano plot (f) of 131 DEmiRNAs. ccRCC: clear cell renal cell carcinoma; DEmRNAs: differentially expressed mRNAs; DElncRNAs: differentially expressed lncRNAs; DEmiRNAs: differentially expressed miRNAs.

The analysis process of our work is illustrated in Figure 1. We integrated the gene expression profiles of 539 ccRCC tissues and 72 normal tissues available from TCGA database into this study. By defining the logFC > 1.0 or <−1.0 and FDR adjusted *P* value < 0.05 as the cut-off, a total of 3075 DEmRNAs (1055 downregulated and 2020 upregulated) and 359 DElncRNAs (71 downregulated and 288 upregulated) were identified between tumor and normal samples (Figures 2(a)–2(d)). We also performed differential expression analysis of miRNA expression profiles between tumor and normal samples to construct the lncRNA-miRNA-mRNA ceRNA network. Consequently, 131 DEmiRNAs (70 downregulated and 61 upregulated) were retrieved with the same filter conditions (Figures 2(e) and 2(f)).

### 3.2. Construction of the ceRNA Network and Survival Analysis

**Figure 3:**
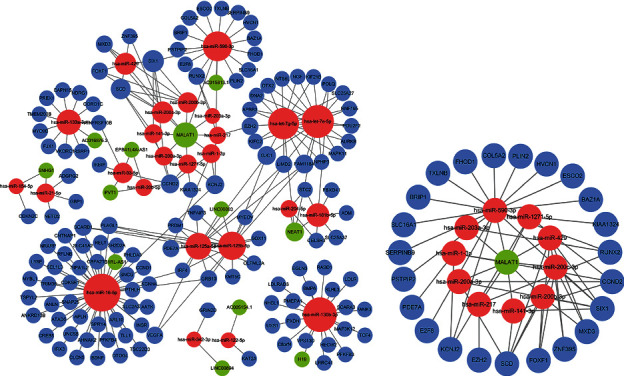
Construction of the ceRNA network via Cytoscape. (a) The ceRNA network of DEmRNAs, DElncRNAs, and DEmiRNAs. (b) The lncRNA MALAT1 subnetwork. Blue circles indicate mRNAs, green balls indicate lncRNAs, and red balls indicate miRNAs.

Based on the 28 pairs of lncRNA-miRNA interactions and 200 pairs of miRNA-mRNA interactions, we constructed a ceRNA network containing 12 DElncRNAs, 25 DEmiRNAs, and 131 DEmRNAs (Figure 3(a)). Afterward, lncRNA MALAT1 and linked miRNAs and mRNAs were included to build a subnetwork (Figure 3(b)). Then, Kaplan-Meier survival analyses were exploited to validate further the association between the genes involved in the ceRNA network and the prognosis of ccRCC. It revealed that a total of 48 genes were markedly related to the overall survival of ccRCC (*P* < 0.001) (Supplementary Table 1).

### 3.3. Functional Enrichment Analysis

**Figure 4:**
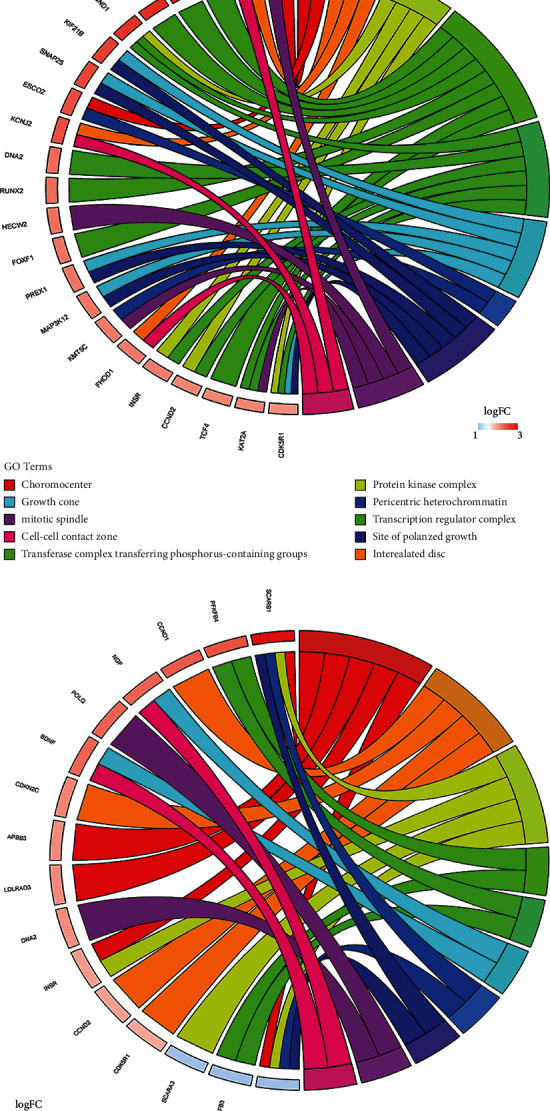
Functional enrichment analysis for DEmRNAs in the ceRNA network. (a–c) The chord diagram of the top 10 significant GO terms and related genes in the cell component (a), biological process (b), and molecular function (c). (d) The bubble plot of the significant KEGG pathways. KEGG: Kyoto Encyclopedia of Genes and Genomes; GO: Gene Ontology.

We performed a functional annotation on 131 DEmRNAs in the ceRNA network. The top 10 enriched GO terms are presented in Figures 4(a), 4(b) and 4(c), including the protein kinase complex and transcription regulator complex in the cell component category, signal transduction by p53 class mediator and positive regulation of the cell cycle process in the biological process category, and cyclin-dependent protein serine/threonine kinase regulator activity and death receptor binding in the molecular function category. The significant KEGG pathways are revealed in Figure 4(d), containing multiple cancer-related pathways, such as the VEGF signaling pathway, Notch signaling pathway, p53 signaling pathway, focal adhesion, and cell cycle.

### 3.4. Construction of the ceRNA-Associated Prognostic Signature

**Figure 5:**
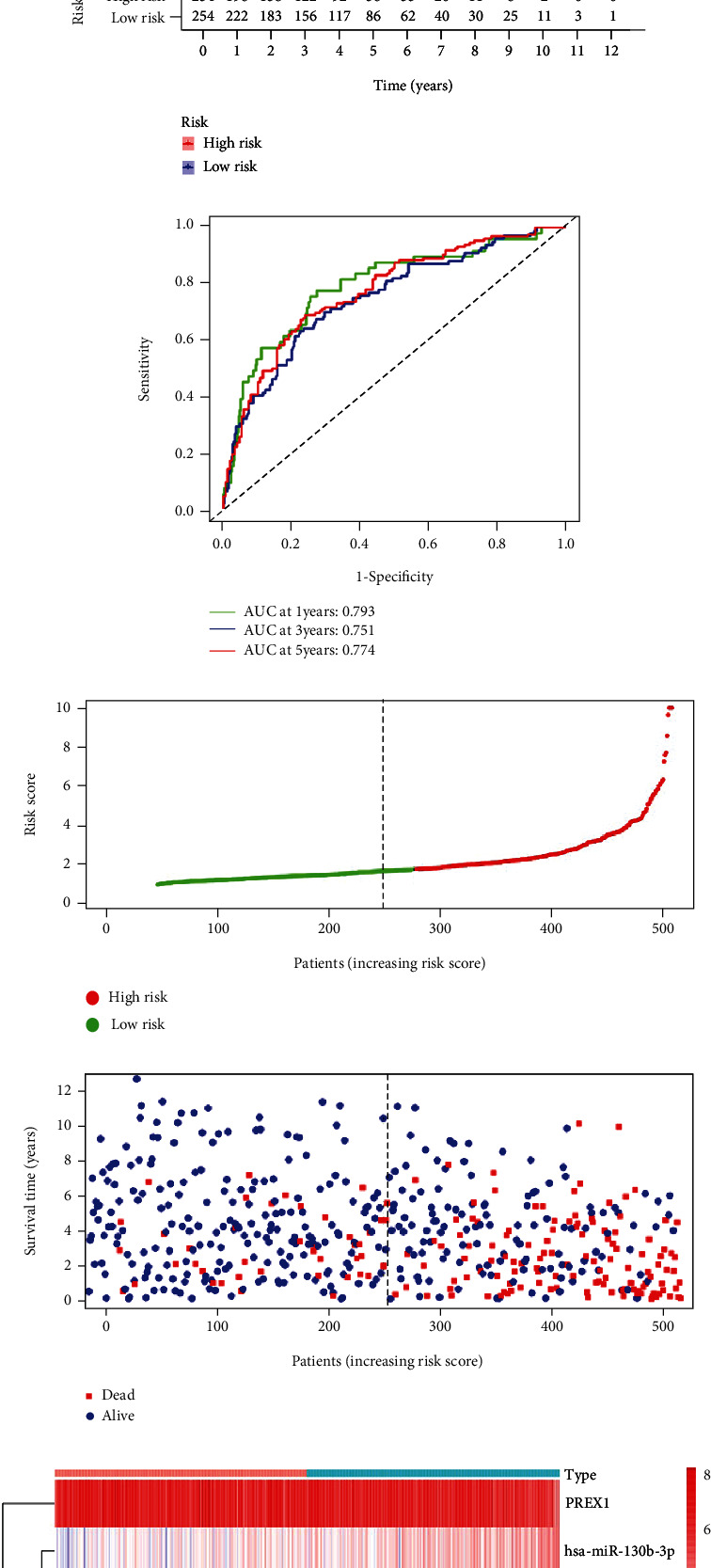
Construction and evaluation of ceRNA-associated prognostic signature for ccRCC. (a, b) Lasso regression analysis retained 18 genes in the ceRNA network. (c) Forest plots indicated that seven genes were incorporated into multivariate Cox regression analysis to predict the survival of ccRCC patients. (d) The K-M survival curve of ccRCC patients in the high- and low-risk groups based on the 7-gene signature. (e) The ROC curves of 1-, 3-, and 5-year OS for the 7-gene signature. (f, g) The scatter plots of risk score distribution and survival state of each patient. (h) The heat map of the expression of seven key prognostic genes in the high- and low-risk groups. (i) The constructed nomogram for predicting the 1-, 3-, and 5-year prognosis of ccRCC patients. (j) The calibration curve for demonstrating the discrimination and accuracy of the nomogram. K-M: Kaplan-Meier; ROC: receiver operating characteristic; OS: overall survival.

Univariate Cox analysis was employed using the R survival package coxph function with a cut-off criterion of *P* value < 0.05. Then, Lasso regression analysis was applied to determine the optimal adjustment parameter through 10-fold cross-validation to avoid overfitting (Figures 5(a) and 5(b)). Ultimately, seven key genes in the ceRNA network, including RELT, CNTNAP1, KIAA1324, PREX1, OTOGL, LINC00894, and miR-130b-3p, were integrated into the ceRNA-related signature for subsequent studies by multivariate Cox regression (Figure 5(c)). In addition, Kaplan-Meier curves were plotted to demonstrate that all seven key genes were significantly associated with survival in ccRCC patients (Supplementary Figure 1). We determined the weight coefficient of each hub gene and calculated the risk score according to the following formula: Risk score = 0.266∗RELT + 0.315∗CNTNAP1 + 0.202∗KIAA1324 − 0.303∗PREX1 − 0.108∗OTOGL + 0.185∗LINC00894 + 0.274∗miR − 130b − 3p (Table 1). The risk score stratified the ccRCC patients into high-risk and low-risk groups based on the median cut-off value. Subsequently, the Kaplan-Meier analysis suggested that the high-risk group had a worse prognosis than the low-risk group (Figure 5(d)), demonstrating that the ceRNA-related prognostic signature owned great predictive value. The ROC curves manifested favorable predictive accuracy in survival outcomes (AUC of 1-year survival: 0.801, AUC of 3-year survival: 0.763, and AUC of 5-year survival: 0.78) (Figure 5(e)). Meanwhile, the risk curve and scatter plot also displayed each individual's risk score and corresponding survival status (Figures 5(f) and 5(g)). The heat map displayed that PREX1 and OTOGL were downregulated in the high-risk group, while miR-130-3p, RELT, CNTNAP1, KIAA1324, and LINC00894 were upregulated in the high-risk group (Figure 5(h)). Based on the ceRNA risk model, the nomogram was generated to predict the 1-, 3-, and 5-survival probability of each patient with ccRCC (Figure 5(i)). Then, the calibration curve indicated good precision and discrimination of the nomogram (Figure 5(j)).

### 3.5. Profiles of TIICs in ccRCC

**Figure 6:**
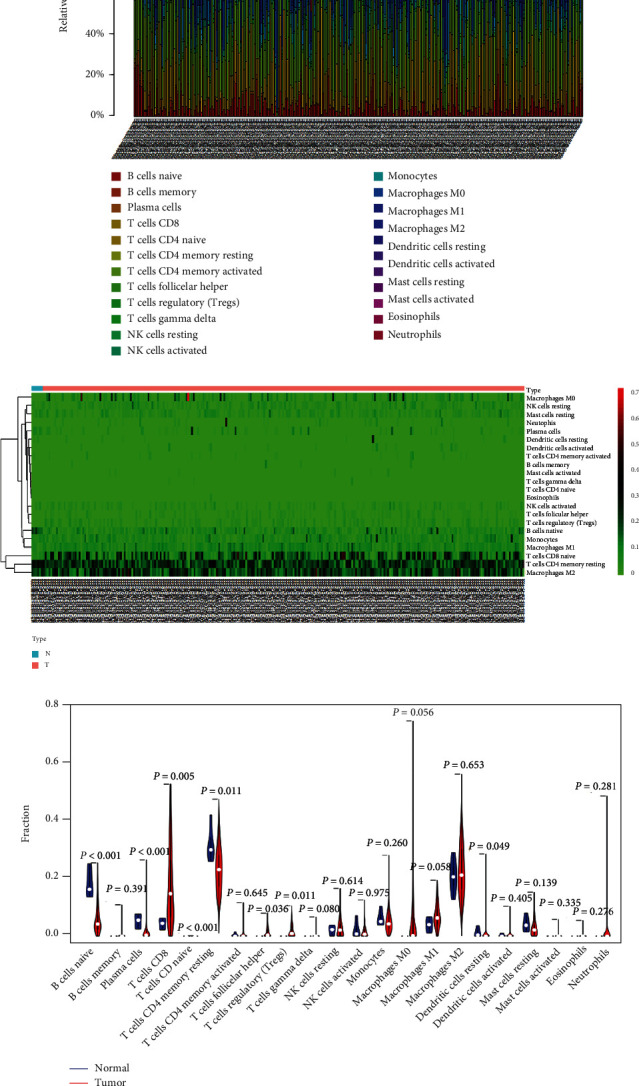
(a, b) The composition (a) and heat map (b) of 22 TIICs assessed by the CIBERSORT algorithm in ccRCC. (c) The violin plot of different infiltration levels of immune cells in tumor and normal groups. TIIC: tumor-infiltrating immune cells; CIBERSORT: cell type identification by estimating relative subsets of RNA transcripts.

The CIBERSORT algorithm was adopted to assess the fractions of 22 TIICs in ccRCC patients, which was presented in the histogram and the heat map (Figures 6(a) and 6(b)). The distribution difference of TIICs between tumor and normal groups was calculated by the Wilcoxon rank-sum test, which depicted that naive B cells, plasma cells, resting memory CD4 T cells, and resting dendritic cells were significantly lower in ccRCC. In contrast, naive CD4 T cells, CD8 T cells, follicular helper T cells, and regulatory T cells were markedly higher (Figure 6(c)).

### 3.6. Construction of the TIIC-Associated Prognostic Signature

**Figure 7:**
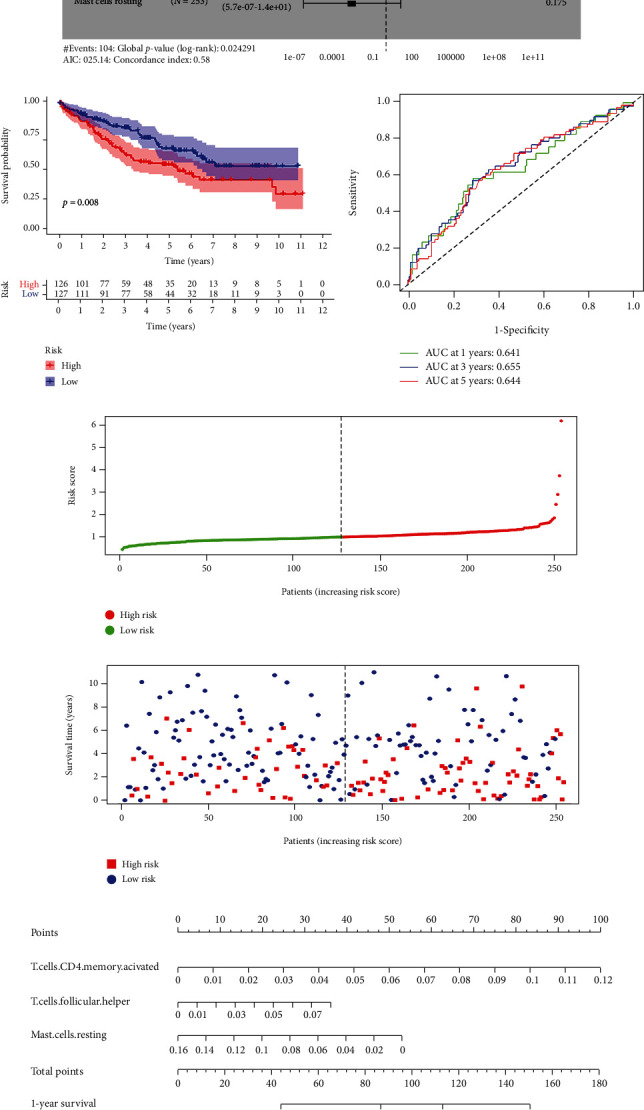
Construction and evaluation of a TIIC-associated prognostic signature for predicting survival risk of ccRCC. (a) The K-M survival curves of the prognostic-related TIICs in ccRCC. (b, c) The results of the Lasso regression analysis suggested that all three TIICs were essential for modeling. (d) Forest plots indicated that three TIICs were incorporated into multivariate Cox regression analysis to predict the survival of ccRCC patients. (e) The K-M survival curve of ccRCC patients in high- and low-risk groups. (f) The ROC curves of 1-, 3-, and 5-year OS for the multivariate Cox model. (g, h) The scatter plots of risk score distribution and survival state of each patient. (i) The heat map of the proportion of the three prognostic TIICs in the high- and low-risk groups. (j) The constructed nomogram for predicting the 1-, 3-, and 5-year prognosis of ccRCC patients. (k) The calibration curve for demonstrating the discrimination and accuracy of the nomogram.

Kaplan-Meier survival analysis was employed to identify the prognosis-related TIICs in ccRCC, which suggested that the infiltration levels of follicular helper T cells and regulatory T cells were significantly associated with survival (Figure 7(a)). Subsequently, 22 TIICs were integrated into 5univariate Cox regression analysis. After Lasso regression and multivariate Cox regression analyses, three TIICs, including activated memory CD4 T cells, follicular helper T cells, and resting Mast cells, constituted a novel TIIC-associated prognostic signature (Figures 7(b), 7(c) and 7(d), Table 2). Further, the survival curve revealed a worse prognosis for patients with high-risk scores (Figure 7(e)). Meanwhile, the ROC curves showed that the prognostic signature manifested acceptable predictive accuracy (AUC of 1-year survival: 0.641, AUC of 3-year survival: 0.655, and AUC of 5-year survival: 0.644) (Figure 7(f)). The risk curve and scatter plot illustrated that the higher the risk scores, the more significant the mortality rate of ccRCC patients (Figures 7(g) and 7(h)). The heat map displayed the infiltration fractions of three immune cells in the high-risk and low-risk groups (Figure 7(i)). The nomogram was then conducted to predict the probability of survival for each patient, and the calibration curve showed good consistency between the nomogram prediction and the actual observation (Figures 7(j) and 7(k)).

### 3.7. Coexpression Analysis

**Figure 8:**
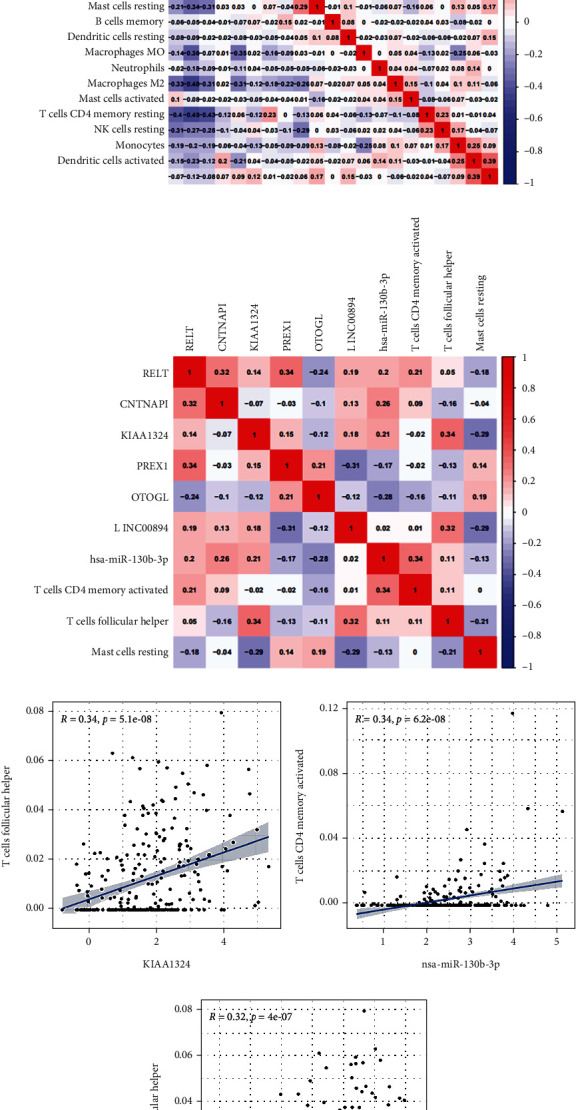
(a) Coexpression analysis. Correlation heat map of proportions of diversified immune cells in ccRCC. (b) Correlation heat map of the hub genes in the ceRNA and the prognostic TIICs. (c–e) KIAA1324 and LINC00894 were positively associated with follicular helper T cells (c, d), and miR-130b-3p was positively associated with activated memory CD4 T cells (e).

The possible correlations among diversified immune cells are shown in Figure 8(a). The coexpression analysis of seven key genes in the ceRNA-related signature with three prognostic-related immune cells in the TIIC-related signature was performed via the Pearson correlation analysis (Figure 8(b)). There were significantly positive correlations between KIAA1324 and follicular helper T cells (*R* = 0.338, *P* < 0.001), LINC00894 and follicular helper T cells (*R* = 0.316, *P* < 0.001), and miR-130b-3p and activated memory CD4 T cells (*R* = 0.336, *P* < 0.001) (Figures 8(c), 8(d) and 8(e)). KIAA1324 was selected for further validation based on correlation coefficient R ranking. We evaluated the relations between KIAA1324 expression and the special surface markers of follicular helper T cells in ccRCC using the TIMER database. The results revealed that CD185 (CXCR5), CD278 (ICOS), CD279/PD-1 (PDCD1), CD3 (CD3D/CD3E/CD3G), CD4, and CXCL13 had significant coexpression patterns with KIAA1324 (Supplementary Figure 2).

### 3.8. Identification of a Potential ceRNA Regulatory Axis

**Figure 9:**
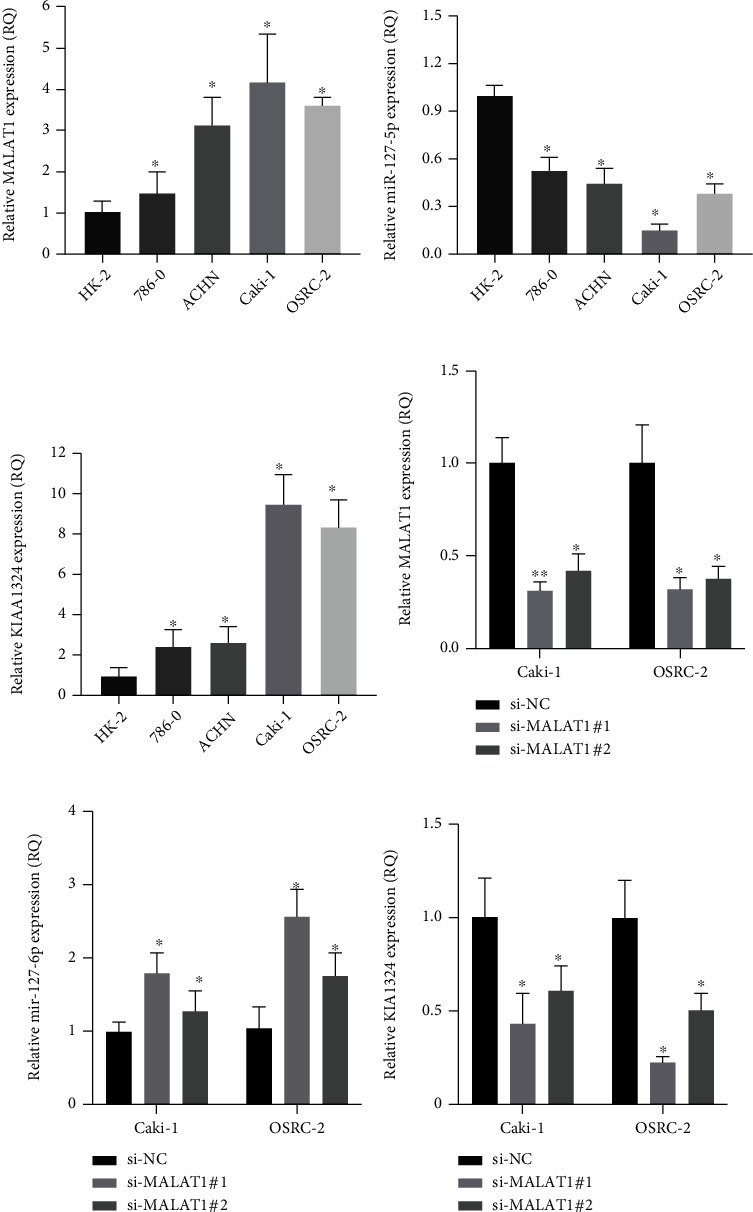
Experimental validation of the MALAT1/miR-1271-5p/KIAA1324 axis. (a–c) MALAT1 and KIAA1324 were upregulated in ccRCC cell lines (86-O, ACHN, Caki-1, and OSRC-2) compared with the normal renal tubular epithelial cell line (HK-2) (a, c), and miR-1271-5p was downregulated in ccRCC cells compared with normal renal tubular epithelial cells (b). (d) The knockdown efficiency of si-MALAT1 in Caki-1 and OSRC-2 cells. (e, f) The expression levels of miR-1271-5p and KIAA1324 after MALAT1 knockdown in Caki-1 and OSRC-2 cells.

Combined with the results above, we identified a potential regulatory axis of MALAT1/miR-1271-5p/KIAA1324 from the ceRNA subnetwork. qRT-PCR was further performed to detect the expression levels of MALAT1, miR-1271-5p, and KIAA1324 in ccRCC cell lines (786-O, ACHN, Caki-1, and OSRC-2) and the normal renal tubular epithelial cell line (HK-2). The results indicated that MALAT1 and KIAA1324 were highly expressed in ccRCC cell lines compared to a normal renal tubular epithelial cell line, while miR-1271-5p was lowly expressed (Figures 9(a), 9(b) and 9(c)). We selected Caki-1 and OSRC-2 cell lines as the MALAT1 overexpresses for MALAT1 knockdown using siRNA (Figure 9(d)). The results showed that the expression of KIAA1324 was markedly downregulated when MALAT1 was silenced in Caki-1 and OSRC-2 cells (Figure 9(e)). In contrast, miR-1271-5p expression was significantly upregulated after MALAT1 silencing (Figure 9(f)).

## 4. Discussion

Clear cell renal cell carcinoma is a common urinary cancer with high morbidity and mortality worldwide and is prone to resistance to chemotherapy and radiotherapy [13]. Compared to conventional treatments, immunotherapy has shown great potential as a novel therapeutic option in the treatment of cancer [14, 15]. However, some patients are unable to benefit from it. Thus, it is essential to explore molecular markers for predicting whether patients could benefit from immunotherapy. Emerging evidence suggests that tumor molecular profiles and the landscape of TIICs play an essential role in tumor development, and they have been proposed as potential prognostic biomarkers [16, 17].

With the rapid development of bioinformatic approaches and high-throughput sequencing technology, researchers have identified an increasing number of abnormally expressed RNAs and differences in the proportions of immune cells between tumor and normal tissues. The construction of ceRNA networks could provide a more comprehensive view of RNA regulatory mechanisms in ccRCC, rather than emphasizing a specific molecular interaction. In the present study, we identified the prognostic-related ceRNAs and TIICs and constructed two prognostic signatures that were confirmed to have good predictive value with high accuracy and discrimination. Based on the level of gene expression signatures and TIICs, we explored the potential regulatory relationship between the MALAT1/miR-1271-5p/KIAA1324 axis and immune responses.

lncRNA is a class of non-protein-coding RNA consisting of more than 200 nucleotides that plays a pivotal role in the development and progression of cancer [18]. The ceRNA hypothesis suggests that lncRNAs may modulate the expression of mRNA by acting as an endogenous sponge to interact with miRNAs, which has been widely accepted in recent years [19]. In the current study, the constructed ceRNA network consisted of 12 DElncRNAs, 25 DEmiRNAs, and 131 DEmRNAs in ccRCC samples according to the transcription profiles from TCGA ccRCC cohort. Furthermore, Gene Ontology (GO) and Kyoto Encyclopedia of Genes and Genomes (KEGG) enrichment analyses were performed to investigate the potential function and pathways of the DEmRNAs. GO analysis revealed that cyclin-dependent protein serine/threonine kinase regulator activity, positive regulation of the cell cycle process, and protein kinase complex were associated with various malignancies [20, 21]. KEGG analysis showed that the DEmRNAs were significantly enriched in the cell cycle, focal adhesion, VEGF signaling, and Notch signaling. Cell cycle and focal adhesion are considered the leading causes of ccRCC initiation and progression [22, 23], while VEGF and Notch signaling pathways have been confirmed to play an important role in the molecular regulation of ccRCC [24, 25]. These might explain that the DEmRNAs in the ceRNA network were significantly correlated with the survival of ccRCC.

We observed that the expression of RELT, CNTNAP1, KIAA1324, PREX1, OTOGL, LINC00894, and hsa-miR-130b-3p in the ceRNA network was significantly related to the survival status of patients with ccRCC. We then constructed a nomogram containing seven genes to accurately predict the prognosis of individual patients. In addition, we employed the CIBERSORT algorithm to identify the different proportions of various TIICs between ccRCC and normal tissues. Similarly, we constructed a prediction model composed of activated memory CD4 T cells, follicular helper T cells, and resting mast cells through a series of statistical analyses, which performed well in predicting the overall survival of ccRCC. Subsequently, we attempted to determine the association of the hub genes in the ceRNA-related signature with the immune cells in the TIIC-related signature. We proposed a ceRNA regulatory axis in which MALAT1 regulated KIAA1324 expression by sponging miR-1271-5p to elucidate the potential mechanism for different degrees of immune infiltration in ccRCC progression. This was preliminarily validated in cell lines by qRT-PCR.

Numerous studies have indicated that MALAT1 has a significant effect on cancer development and progression. MALAT1 is implicated in the regulation of several signaling pathways such as ERK/MAPK [25], Notch [26], and Wnt/*β*-catenin [27], affecting cell proliferation, apoptosis, migration, invasion, immunity, and inflammatory response. Moreover, MALAT1 has been reported to modify ccRCC progression via regulating miR-194-5p/ACVR2B signaling [28]. Previous studies have suggested that miR-1271-5p is closely associated with the progression of several malignancies. Han et al. found that miR-1271-5p suppressed the progression of ovarian cancer through Notch signaling [29]. Furthermore, it was reported that MALAT1 sponges miR-1271-5p and facilitates multiple myeloma and ovarian cancer progression by regulating SOX13 and E2F5, respectively [30, 31]. In gastric cancer, Kang et al. demonstrated that KIAA1324 was downregulated and suppressed cell proliferation, invasion, and chemoresistance [32]. However, Schlumbrecht et al. indicated that overexpression of KIAA1324 was correlated with worse prognosis in high-grade serous carcinoma of the ovary/peritoneum [33]. The role of KIAA1324 in cancer has not yet been evaluated in ccRCC. According to the functional enrichment analysis results, we hypothesized that MALAT1 might regulate the expression of KIAA1324 as a miRNA sponge for miR-1271-5p to regulate the Notch signaling pathway in ccRCC. KIAA1324 may serve as a potential prognostic marker for patients with ccRCC and predict the benefit of immunotherapy. In future studies, we will further focus on validating this molecular mechanism in vitro and in vivo.

In the current study, coexpression analysis demonstrated that KIAA1324 expression was positively related to follicular helper T cells (*R* = 0.338, *P* < 0.001). As expected, we found that KIAA1324 expression was positively associated with most follicular helper T cell-related markers. Follicular helper T cells are a predominant subset of CD4+ T cells specialized in providing requisite help for B cells to promote the formation of germinal centers and generate high-affinity antibodies [34, 35]. Accumulating evidence has suggested that dysregulation of follicular helper T cells is involved in various diseases. Numerous studies have demonstrated that the infiltration of follicular helper T cells in multiple malignancies is positively associated with survival, including that of non-small-cell lung cancer [36], colorectal cancer [37], and breast cancer [38]. Furthermore, Bronsert et al. reported that high numbers and densities of PD1+ follicular helper T cells were linked to adverse prognosis in triple-negative breast cancer [39]. Taken together, we speculated that the MALAT1/miR-1271-5p/KIAA1324 axis is closely related to the poor prognosis of ccRCC by regulating follicular helper T cell infiltration.

We comprehensively elucidated the potential molecular mechanism for the progression of immune infiltration in ccRCC by constructing a ceRNA regulatory network and axis. Nevertheless, we have to acknowledge that there are some limitations to the current study. First, this is a retrospective study based on the public database with a relatively small normal sample size and incomplete clinical information. Furthermore, due to the multiple sources of public data, further confirmation is needed with homogeneous study samples as well as systematic experiments. Second, the data used in our study mainly derive from Western countries, so the results are applied with caution in Asian populations. Third, in the CIBERSORT analysis, we only took into account the proportion of TIICs and ignored the heterogeneity of the immune microenvironment related to the location of immune infiltration. However, in spite of its limitations, our study innovatively proposed that MALAT1, miR-1271-5p, KIAA1324, and follicular helper T cells might be closely associated with the progression of ccRCC. Further biological studies should be conducted to validate our results.

## 5. Conclusion

In summary, based on ceRNA network construction and immune infiltration analysis, we identified two prognostic signatures with good predictive value and utility for assessing the survival of ccRCC patients. In addition, our study inferred that the MALAT1/miR-1271-5p/KIAA1324 axis and the infiltration of follicular helper T cells might play critical roles in the progression of ccRCC. KIAA1324 could be used as a potential prognostic marker for predicting the benefit of immunotherapy in patients with ccRCC. Due to the lack of experimental validation in vitro and in vivo, the mechanism of KIAA1324-induced immune infiltration still requires further investigation.

## Figures and Tables

**Table 1:** Multivariate Cox regression analysis identifying seven key genes of the ceRNA network for prognosis in ccRCC.

Gene	Coefficients	HR	95% CI lower	95% CI upper	*P* value
RELT	0.27	1.30	1.01	1.68	0.039
CNTNAP1	0.32	1.37	1.14	1.65	0.001
KIAA1324	0.20	1.22	1.09	1.38	0.001
PREX1	-0.30	0.74	0.61	0.90	0.003
OTOGL	-0.11	0.90	0.79	1.02	0.088
LINC00894	0.19	1.20	1.07	1.35	0.002
miR-130b-3p	0.27	1.32	1.08	1.60	0.006

**Table 2:** Multivariate Cox regression analysis identifying three key TIICs for prognosis in ccRCC.

Immune cells	Coefficients	HR	95% CI lower	95% CI upper	*P* value
T cells CD4 memory activated	14.78	2.62*E* + 06	7.48	9.15*E* + 11	0.023
T cells follicular helper	7.99	2.96*E* + 03	0.08	1.13*E* + 08	0.137
Mast cells resting	-5.88	2.79*E* − 03	5.72*E* − 07	13.66	0.174

## Data Availability

The data and materials supporting the findings of this study are available from the corresponding author upon reasonable request.

## Supplementary Materials

Supplementary Materials Supplementary Figure 1: (a–g) Kaplan-Meier survival curves of seven key genes in the ceRNA network of ccRCC. Supplementary Figure 2: the evaluation of relationship with KIAA1324 of the specific surface markers of follicular helper T cells. (a, b) The surface markers of follicular helper T cells, reported more than twice in previous studies, were acquired from the CellMarker database. (c) KIAA1324 expression was positively correlated with most surface markers of the follicular helper T cell. Supplementary Table 1: survival analysis of 48 genes in the ceRNA network of ccRCC. Supplementary Table 2: primer sequences used in qRT-PCR.
