# Supplementary material for: Identification of Novel Prognostic Signatures for Clear Cell Renal Cell Carcinoma Based on ceRNA Network Construction and Immune Infiltration Analysis
Source: Dis Markers. 2022 Mar 14;2022:4033583. doi: 10.1155/2022/4033583 (PMC8938059; doi:10.1155/2022/4033583)
Supplement: Supplementary Materials — Supplementary Figure 1: (a–g) Kaplan-Meier survival curves of seven key genes in the ceRNA network of ccRCC. Supplementary Figure 2: the evaluation of relationship with KIAA1324 of the specific surface markers of follicular helper T cells. (a, b) The surface markers of follicular helper T cells, reported more than twice in previous studies, were acquired from the CellMarker database. (c) KIAA1324 expression was positively correlated with most surface markers of the follicular helper T cell. Supplementary Table 1: survival analysis of 48 genes in the ceRNA network of ccRCC. Supplementary Table 2: primer sequences used in qRT-PCR. [file 4033583.f1.docx]

**Supplementary Materials**

**Figure Legends**

SUPPLEMENTARY FIGURE 1: (a-g) Kaplan-Meier survival curves of seven key genes in the ceRNA network of ccRCC.

SUPPLEMENTARY FIGURE 2: The evaluation of relationship with KIAA1324 of the specific surface markers of follicular helper T cells. (a and b) The surface markers of follicular helper T cells, reported more than twice in previous studies, were acquired from the CellMarker database. (c) KIAA1324 expression was positively correlated with most surface markers of follicular helper T cell.

SUPPLEMENTARY TABLE 1. Survival analysis of 48 genes in the ceRNA network of ccRCC.

| Gene | *P*-value |
| --- | --- |
| CELSR3 | 0.000721661 |
| ANLN | 0.000720923 |
| SH2D2A | 2.66E-05 |
| POU2F2 | 0.00035887 |
| TLL1 | 3.28E-05 |
| VPS13D | 7.51E-10 |
| POLQ | 4.45E-05 |
| RELT | 0.000344427 |
| DTX2 | 7.13E-05 |
| KCNN4 | 1.58E-09 |
| GRIN2D | 6.94E-07 |
| GRB10 | 0.000128697 |
| EZH2 | 0.000468629 |
| KAT2A | 6.51E-08 |
| CNTNAP1 | 7.99E-09 |
| CCND1 | 0.000116561 |
| APBB3 | 2.09E-05 |
| NPHP3 | 3.59E-05 |
| KIAA1324 | 4.81E-06 |
| PREX1 | 1.30E-05 |
| KMT5C | 8.91E-07 |
| LIMD2 | 1.47E-05 |
| DNA2 | 6.07E-07 |
| HECW2 | 3.01E-06 |
| MAP3K12 | 6.45E-08 |
| SLC25A37 | 6.99E-06 |
| PLIN2 | 0.0001304 |
| TRIM36 | 0.000312122 |
| BMP6 | 0.000285069 |
| INTS6L | 0.000806035 |
| OTOGL | 6.39E-06 |
| KIFC2 | 0.000162479 |
| INSR | 0.000295396 |
| CLCN5 | 4.36E-08 |
| ATAD5 | 0.000288388 |
| AURKB | 1.43E-07 |
| AC005154.1 | 2.39E-06 |
| ANKRD13B | 0.000263123 |
| MXD3 | 1.06E-10 |
| EPB41L4A-AS1 | 0.000134658 |
| LINC00894 | 0.000407554 |
| LINC00893 | 0.000615801 |
| PVT1 | 2.48E-05 |
| AC015813.1 | 1.28E-05 |
| AC016876.2 | 0.000712736 |
| hsa-miR-130b-3p | 4.83E-06 |
| hsa-miR-204-5p | 9.00E-05 |
| hsa-miR-21-5p | 6.70E-07 |

SUPPLEMENTARY TABLE 2: Primer sequences used in qRT-PCR.

| Gene | Forward primer (5'-3') | Reverse primer (5'-3') |
| --- | --- | --- |
| MALAT1 | CAGGTCGTGTATCTCCGCCA | TCATACTACCAGTCTCACAC |
| KIAA1324 | CAAGCCTGTGCTGACACAAC | GCCTGTCATGCCCTTGTACT |
| miR-1271-5p | CTTGGCACCTAGCAAGCACTCA | GCGAGCACAGAATTAATACGAC |
